# Pseudocystic myxoid liposarcoma of the posterior chest wall – a rare case report

**DOI:** 10.1097/MS9.0000000000001431

**Published:** 2023-11-01

**Authors:** Saad Shakil, Sareema Eman Akhtar, Mahum Zaidi, Rizwan Ajmal, Abdulla K. Alsubai, Rahul Ramtohul, Absam Akbar, Aysa Tabassi, Aylin Tabassi, Talal Almas

**Affiliations:** a Internal Medicine, Ziauddin Medical College; b Radiology Department; c Liaquat National Hospital and Medical College; d Aga Khan University Hospital, Karachi, Pakistan; e Beaumont Hospital; f RCSI University of Medicine and Health Sciences, Dublin, Ireland; g University Hospitals Cleveland Medical Center, Cleveland, Ohio, USA

**Keywords:** liposarcoma, myxoid, posterior chest wall, pseudocystic, rare location

## Abstract

**Introduction and importance:**

Liposarcoma (LPS) is a common soft-tissue sarcoma predominantly diagnosed in adults, arising from malignant adipose cells. Among its various subtypes, myxoid LPS (MLPS) stands out as the second most frequent, accounting for ~30% of all LPS cases. This particular subtype typically manifests in males between the ages of 40 and 50 and is commonly found in the lower extremities. Although rare, MLPS may also occur in the head, neck, and infrequently in the back. Chest wall LPS cases are also sparsely reported.

**Case presentation:**

In this report, we present a case of MLPS in a 69-year-old male patient who presented with a complaint of firm swelling on the right posterior chest wall, which was progressively increasing in size over the past 10 years. The tumour was located in the posterior chest wall on the left side, and further diagnostic evaluation using computed tomography (CT) and MRI was conducted to identify its characteristics and extent.

**Clinical discussion:**

The use of CT scanning plays a crucial role in differentiating between various lipomatous tumour types, aiding in the identification and classification of MLPS. However, MRI emerges as a more effective technique for detecting microscopic fat compared to CT or ultrasonography, providing valuable insights for accurate diagnosis and treatment planning.

**Conclusion:**

Surgery remains the primary therapeutic approach for managing LPSs, including MLPS. Adjuvant preoperative radiation is recommended due to its significant sensitivity and potential for improved outcomes. Given the rarity of this presentation and the varied anatomical locations, a multidisciplinary approach is paramount in effectively managing such cases. Medical practitioners should collaborate closely, considering the unique challenges posed by MLPS to ensure optimal patient care and treatment outcomes.

## Introduction

Highlights

- The study presents a rare case of myxoid liposarcoma (MLPS), which is extremely unusual for this subtype of liposarcoma (LPS) to occur in the posterior chest wall.
- A 69-year-old male with a decade-long history of hypertension and diabetes mellitus presented with a gradually enlarging firm swelling on the right posterior chest wall.
- The differential diagnosis for the firm swelling on the posterior chest wall that led to suspicion of a neoplastic lesion on ultrasonography is LPS or myxofibrosarcoma.
- Preoperative assessment of lipomatous and myxoid tumours often involves computed tomography scanning and MRI, with MRI being better at identifying microscopic fat and distinguishing LPS from other soft-tissue lesions.
- Surgery is the mainstay treatment for LPSs, with wide and deep surgical excision recommended for high-grade lesions. Preoperative radiotherapy is commonly used, while chemotherapy may have limited impact, especially for larger tumours.
- The exceptional rarity of this presentation underscores the imperative for a multidisciplinary approach among medical professionals to effectively manage such cases.

The most prevalent soft-tissue sarcoma in adults is liposarcoma (LPS), which is often a cancer of adipose cells. In a middle-aged individual, LPS often presents as a slowly growing, painless, non-ulcerated submucosal lump. Nevertheless, some lesions grow quickly and develop ulcers before others do^1^. Soft-tissue tumours comprise 50% of benign lipomas and around 1% of malignant sarcomas of soft tissue. These rare LPSs occurring in the adult population report a frequency of less than 1% and an incidence of less than 30 cases per million people. The location of the tumour, and more specifically, its histological pattern, has a significant impact on the tumour’s prognosis^2^.

LPS is classified into four main groups: Well-differentiated LPS (WDLPS; also known as atypical lipomatous tumour), dedifferentiated LPS (DDLPS), myxoid LPS (MLPS), and pleomorphic LPS^3^. MLPS is the second most prevalent type of LPS, contributing to around 30% of all LPS cases and is usually seen in the fourth and fifth decades of life, commonly manifesting in males^4,5^. This particular subtype frequently develops in the lower extremities^6^. They are occasionally found in the head and neck and seldom in the back^7^.

In the case presented, a MLPS was identified, which on computed tomography (CT) scan reporting was classified as a pseudocystic LPS. This case is particularly remarkable due to the location of the LPS, which is very unusual as it is uncommon to find this tumour in the back. The present case was reported in line with the SCARE 2020 criteria^8^.

## Case report

A 69-year-old male with a known case of hypertension and diabetes mellitus presented to the outpatient department with a complaint of firm swelling on the right posterior chest wall, which has been progressively increasing in size for the past 10 years. The patient reported that the swelling was not painful but occasionally caused discomfort while lying down.

On examination, the swelling was soft, non-mobile, and non-tender. Fluctuation and transillumination tests were negative. The overlying skin was unremarkable. Further evaluation revealed that the swelling had a well-defined border and was ~8 cm in diameter. The patient denied any history of trauma or previous surgeries in the area.

## Investigations

The patient was advised an ultrasound of soft tissue, which revealed a well-defined hypoechoic lesion in the subcutaneous tissue of the left side of the back, showing arterial and venous flow on colour Doppler imaging, it approximately measured 9.3×7.0 cm, and raised suspicion of neoplastic lesion likely LPS. A CT scan of the back with contrast was advised, along with a biopsy for further evaluation.

CT scan revealed a fluid density cystic area measuring about 9.8×8.8×3.8 cm (LS×TS×AP) (length, transverse, and anteroposterior) involving the subcutaneous tissue of the right posterior chest wall extending up to the midline. There was also mild adjacent fat streaking. It appeared inseparable from underlying posterior spinal muscles. A lymph node measuring 1.0×0.5 cm was seen in the paratracheal region. Included sections from the upper abdomen showed a calcified area in segment VIII of the right lobe of the liver, likely representing an old healed abscess or hydatid cyst. Furthermore, a trucut biopsy was taken and cores were sent to histopathology for a conclusive diagnosis.

## Histopathology

### Gross description

Multiple greyish-white liner cores measuring 0.5 cm in aggregate.

### Microscopic examination

Sections examined revealed neoplastic lesions composed of atypical spindle cells with mild to moderate nuclear pleomorphism set against a loose myxoid background. Few atypical lipoblasts-like cells were also appreciated with hyperchromatic indented nuclei and clear multi-vacuolated cytoplasm.

### Swelling on the back (Trucut biopsy)

Malignant neoplasm (sarcoma). Differential diagnosis includes LPS and myxofibrosarcoma in order of preference.

The patient underwent surgical excision of the tumour, which revealed a large multi-lobulated cystic tumour arising from the muscles of the posterior chest wall, post-operative histopathological examination confirmed a MLPS. The patient continues to do well to date, with regular 6 monthly follow-ups scheduled to monitor the patient.

**Figure 1:**
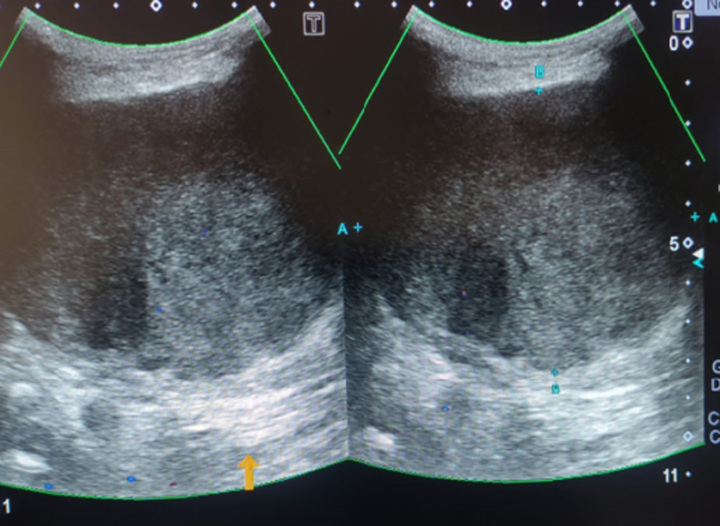
Ultrasound showing a well-defined hypoechoic lesion in the subcutaneous tissue of the left side of the back, approximately measuring 9.3×7.0 cm.

**Figure 2:**
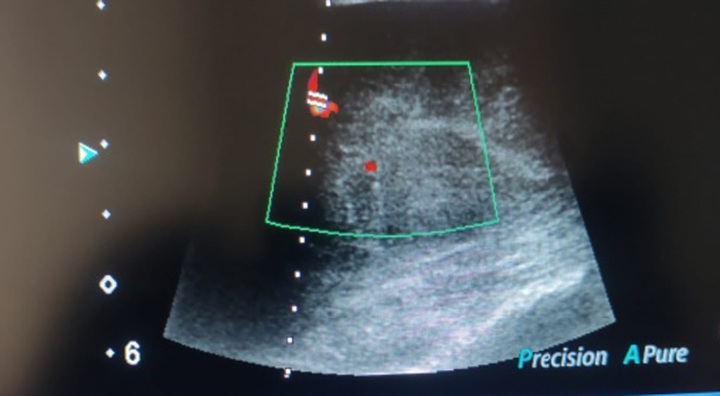
Venous flow within it on colour Doppler imaging.

**Figure 3:**
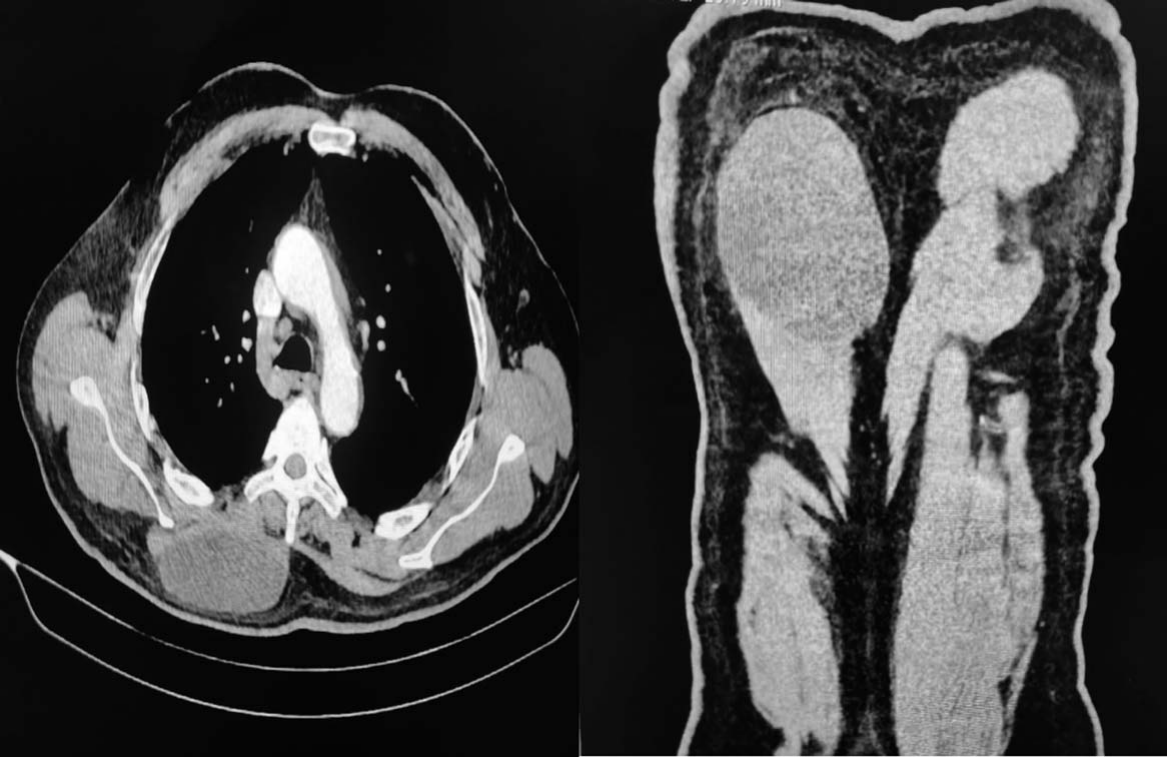
Computed tomography scan of the chest with intravenous contrast shows a fluid density cystic area measuring about 9.8×8.8×3.8 cm (LS×TS×AP) (length, transverse, and anteroposterior) involving the subcutaneous tissue of the right posterior chest wall extending up to the midline. It is associated with mild adjacent fat streaking. It appears inseparable from underlying posterior spinal muscles.

Figures 1–3 show the radiological findings of this case.

A copy of the written consent is available for review by the Editor-in-Chief of this journal on request.

## Discussion

One of the most prevalent malignant soft-tissue sarcomas in adulthood is the LPS, with around 13–60% originating in the thighs and less frequently in the retroperitoneum with no specific gender predisposition. This fat tumour, which is localised everywhere, typically presents as a slowly growing lump with a deceptively innocuous look^9^. It varies from highly cellular or pleomorphic malignant neoplasms to well-differentiated lipoma-like and myxoid tumours. The diverse microscopic image is reflected in the variable clinical behaviour^10^.

LPS often manifests clinically as a painless soft-tissue tumour, similar to other kinds of soft-tissue sarcomas. Meanwhile, 10–15% of instances of LPS are painful^11^. The symptoms of pain typically arise when the tumour becomes large enough to have an impact on surrounding structures. Many patients present with oedema, loss of sensations, and tenderness due to nerve impingement^12^. In more than 90% of instances, MLPS is linked to a chromosomal translocation, most frequently t (12;16) (q13; p11), which causes the DDIT3 and FUS genes to fuse, leading to the production of the FUS-DDIT3 fusion protein^13^. Extrapulmonary areas, notably soft tissue and skeletal locations like the mediastinum and the retroperitoneum, are where MLPS tends to metastasize. Hence, it is crucial to make an early diagnosis and offer the best treatment choices since the disease has the potential to spread to distant places^4^. The chest wall LPS has been documented in a very small number of instances. These included two cases covered in subcutaneous tissue, one with a partly extended latissimus dorsi flap and one in the intermuscular area. Since LPSs often form in the intermuscular fascia, they frequently manifest on or underneath muscles^7^.

Preoperative assessment of soft-tissue lipomatous and myxoid tumours often entails the use of CT scanning. In addition to offering useful morphologic details, CT scanning aids in the distinction between various varieties of lipomatous tumours^14^. On a CT scan, the LPS can have a pure fat, mixed, or pure fluid density, as seen in our case, which is very rare to find. Compared to CT or ultrasound, MRI is better capable of identifying microscopic fat^15^. By identifying an adipose component frequently found in these tumours, MRI with fat-saturated T1-weighted images and gadolinium-enhanced sequences is a useful tool for differentiating LPS from other kinds of soft-tissue lesions^11^. However, in some cases, chest X-rays, CT scans, or even PET scans, which have been shown effective in other soft-tissue sarcomas, may not be able to detect metastatic malignancy in MLPS^16^. To this date, the mainstay treatment of LPSs remains surgery. For high-grade lesions, wide and deep surgical excision, concomitant radiation, and/or chemotherapy may be required^12^. Due to its excellent sensitivity, adjuvant preoperative radiotherapy is recommended. Nonetheless, chemotherapy may have little impact, particularly in tumours bigger than 10 cm^17^.

## Conclusion

Radiologists frequently have the challenge of identifying a deep soft-tissue mass. It is imperative for radiologists to provide a precise and timely preoperative diagnosis and determine the tumour’s extent in order to treat the sarcoma effectively. The very uncommon nature of this presentation highlights the need for medical professionals to address instances like this in a multidisciplinary manner.

## Ethical approval

Ethics approval was not required for this case report as it does not constitute research according to the policies and guidelines of our institution. Institute: Liaquat National Hospital and Medical College, Karachi, Pakistan.

## Consent

Written informed consent was obtained from the patient for publication and any accompanying images. A copy of the written consent is available for review by the Editor-in-Chief of this journal on request.

## Sources of funding

Not applicable/nothing to declare.

## Author contribution

S.S. and S.E.A.: literature search and writing of manuscript introduction and discussion; M.Z. and R.A.: study design and writing of case report body and also reviewed the article; A.K.A., R.R., A.A., and T.A.: reviewed and supervised the article.

## Conflicts of interest disclosure

Not applicable/nothing to declare.

## Research registration unique identifying number (UIN)

Not applicable.

## Guarantor

Talal Almas, MD.

## Data availability statement

Not applicable to this article.

## Provenance and peer review

Not commissioned, externally peer-reviewed.

## Disclosures

Not applicable.
